# Unraveling the efficiency-limiting morphological issues of the perylene diimide-based non-fullerene organic solar cells

**DOI:** 10.1038/s41598-018-21162-x

**Published:** 2018-02-12

**Authors:** Ranbir Singh, Sanjaykumar R. Suranagi, Jaewon Lee, Hansol Lee, Min Kim, Kilwon Cho

**Affiliations:** Department of Chemical Engineering, Pohang University of Science and Technology, Pohang, 37673 Korea

## Abstract

Herein we report a comparative morphological analysis of the perylene diimide (PDI)- and fullerene-based organic solar cells (OSCs) to identify the factors responsible for low performance of PDI-based devices. A PDI derivative, bis-PDI, and a fullerene derivative, PC_70_BM, are mixed with an efficient polymer donor, PffBT4T-2OD. The large disparity in power conversion efficiencies (PCEs) of OSCs composed of PffBT4T-2OD:bis-PDI (PCE = 5.18%) and PffBT4T-2OD:PC_70_BM (PCE = 10.19%) observed are attributed to differences in the nanostructural motif of bulk heterojunction (BHJ) morphologies of these blend systems. The X-ray scattering and surface energy characterizations revealed that the structurally dissimilar bis-PDI and PC_70_BM molecules determine the variation in blend film morphologies, and in particular, the molecular packing features of the donor PffBT4T-2OD polymer. In addition, high-resolution transmission electron microscopy (HRTEM) images explore the BHJ morphologies and presence of longer polymer fibrils in PffBT4T-2OD:bis-PDI system, justifying the unbalanced charge transport and high hole mobility. The low performance of PffBT4T-2OD:bis-PDI devices was further investigated by studying charge carrier recombination dynamics by using light-intensity-dependent and transient photovoltage (TPV) experiments. Furthermore, the temperature-dependent experiments showed the photovoltaic properties, including charge recombination losses, are strongly affected by energetic disorder present in bis-PDI-based system.

## Introduction

Bulk heterojunction (BHJ) OSCs are being extensively researched as a clean energy source with efficiencies exceeding 10%^1–5^. In most of the high-efficiency BHJ-OSCs, the fullerene derivative PC_70_BM is a common constituent, which is used as an electron accepting material in the photoactive layer. Despite the beneficial properties of fullerenes in the context of BHJ-OSCs, they are limited in (i) spectral bandwidth, (ii) ambient stability, (iii) tunability of electronic properties, and (iv) relatively high cost. For these reasons, it is not clear whether fullerene derivatives are ultimately the best candidate materials for use as OSC acceptors. Several research groups have focused on the development of non-fullerene small molecule acceptors. To date, numerous small molecule non-fullerene acceptors have been designed and found to provide PCEs that are comparable to those achieved using fullerene derivatives^4,6–8^. One class of non-fullerene electron accepting materials is low-cost PDI derivatives^7,9–13^. The BHJ photoactive layer made with PDIs offers a high absorption coefficient in the visible region, good photochemical stability, and good thin film forming properties^7,9,14,15^. Despite their attractive features, PDI-based acceptors have met with little success, and few PDI systems have shown PCEs just over 8%^16,17^. One of the reason for having lower PCEs is poor charge carrier transport properties in BHJ blend films, which is mainly depend on the molecular interconnectivity, packing and orientation in blend films.

The bulk characteristics of the photoactive layer of BHJ OSCs depend on the formation of nonbonding intermolecular interactions that facilitate the formation of specific molecular orders, packing structures, and morphologies in the solid state. Unfortunately, in polymer donor:monomeric-PDI acceptor based systems, large-scale phase separation induced by strong molecular aggregation among monomeric PDIs can seriously limit excimers^7^ dissociation and charge transport^18,19^. The molecular aggregation among PDIs was recently ameliorated through the design of twisted and three-dimensional (3D) structured PDI molecules, but still, there are few factors that are limiting the PCEs of the PDI based OSCs as compared to fullerene-based OSCs^20–24^. Therefore, a thorough comparative analysis of the molecular structures of small molecule acceptors and their solid-state properties is necessary for further development of PDI-based acceptors.

In this work, we have studied the morphological and photovoltaic properties of a PDI derivative (bis-PDI) and compared with well-known fullerene-based acceptor (PC_70_BM), combined with donor polymer, poly[(5,6-difluoro-2,1,3-benzothiadiazol-4,7-diyl)-alt-(3,3′′′-di(2-octyldodecyl)-2,2′;5′,2″;5″,2′′′-quaterthiophen-5,5′′′-diyl)] (PffBT4T-2OD). UV-vis absorption and photoluminescence (PL) studies were conducted to characterize the photon harvesting and excited state dissociation parameters. Atomic force microscopy (AFM), HRTEM, grazing incidence wide angle X-ray scattering (GIWAXS), and contact angle measurements were employed to monitor the differences in the film morphologies. TPV study, photocurrent-effective voltage analysis, and low temperature-dependent characterizations were performed to elucidate the factors that determined the differences among device performances.

## Results and Discussion

**Figure 1:**
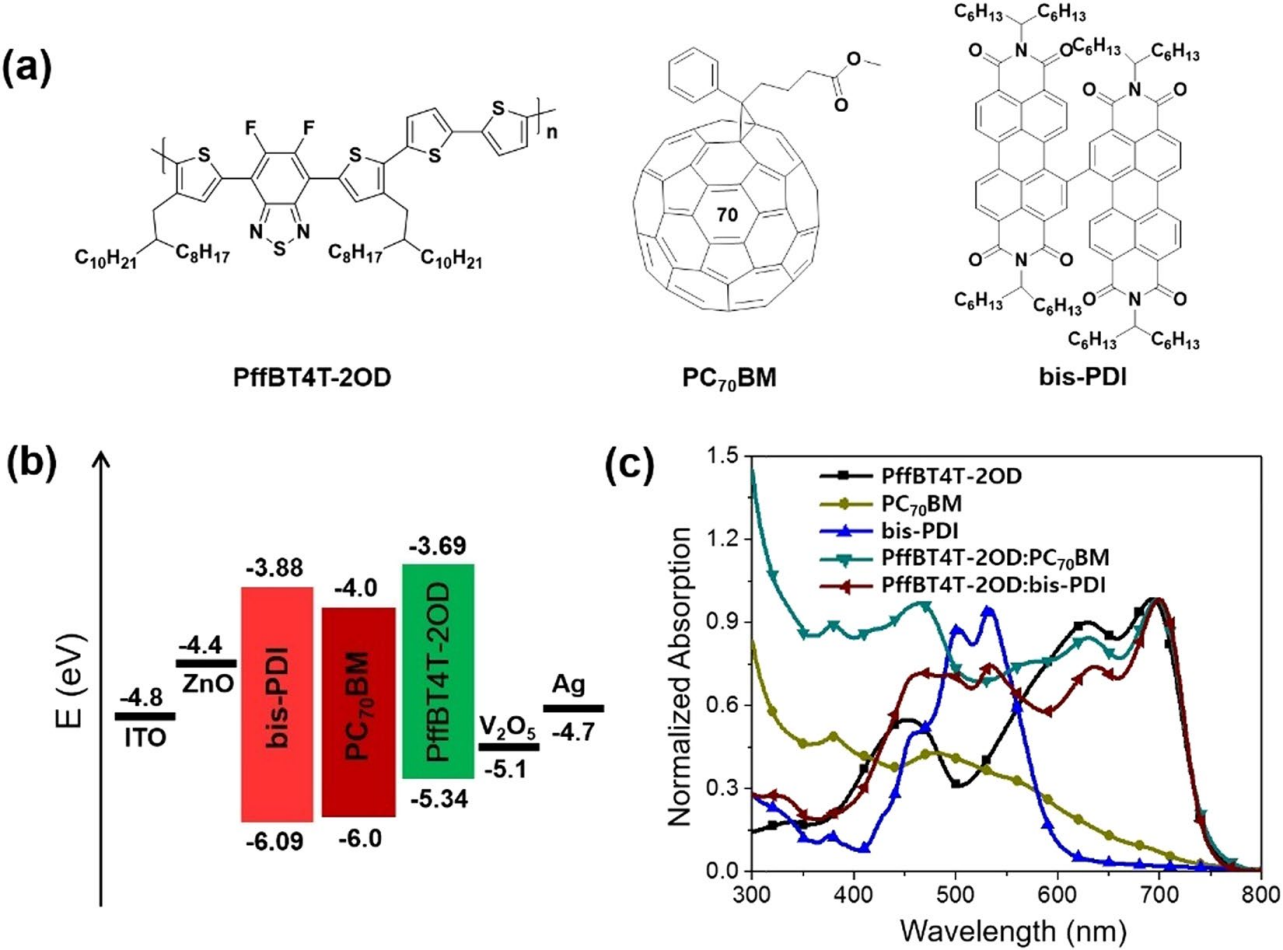
(**a**) Chemical structures of PffBT4T-2OD polymer, PC_70_BM and bis-PDI small molecules, (**b**) energy level diagram of the inverted device structure with active materials used, (**c**) normalized UV-vis absorption spectra for the PffBT4T-2OD polymer, PC_70_BM, bis-PDI, PffBT4T-2OD:PC_70_BM and PffBT4T-2OD:bis-PDI thin films.

The chemical structures and energy levels (HOMO and LUMO) of PffBT4T-2OD, PC_70_BM, and bis-PDI are shown in Fig. 1a,b. PffBT4T-2OD is a crystalline polymeric donor with a broad absorption spectrum in visible region, and OSC devices reported with fullerene acceptors exhibited PCE of 10.8%, one of the highest PCE values recorded for single OSC^25^. The bis-PDI small molecule was synthesized by a modified procedure as described in the supporting information, and used as an acceptor material because of its strong light absorption in visible region (420–600 nm), good electron mobility, and favorable energy level matching with PffBT4T-2OD donor polymer. Figure 1c presents the UV-vis absorption spectra of the pristine (bis-PDI, PC_70_BM and PffBT4T-2OD) and blend (PffBT4T-2OD:PC_70_BM and PffBT4T-2OD:bis-PDI) films, where both blend films exhibit good light harvesting in the visible region (400–780 nm). In addition, PffBT4T-2OD:bis-PDI blend film shows a weak absorption near-UV region (300–420 nm), this difference might be attributed to weak absorption of bis-PDIs in this region.

### Solar cell device performance

**Figure 2:**
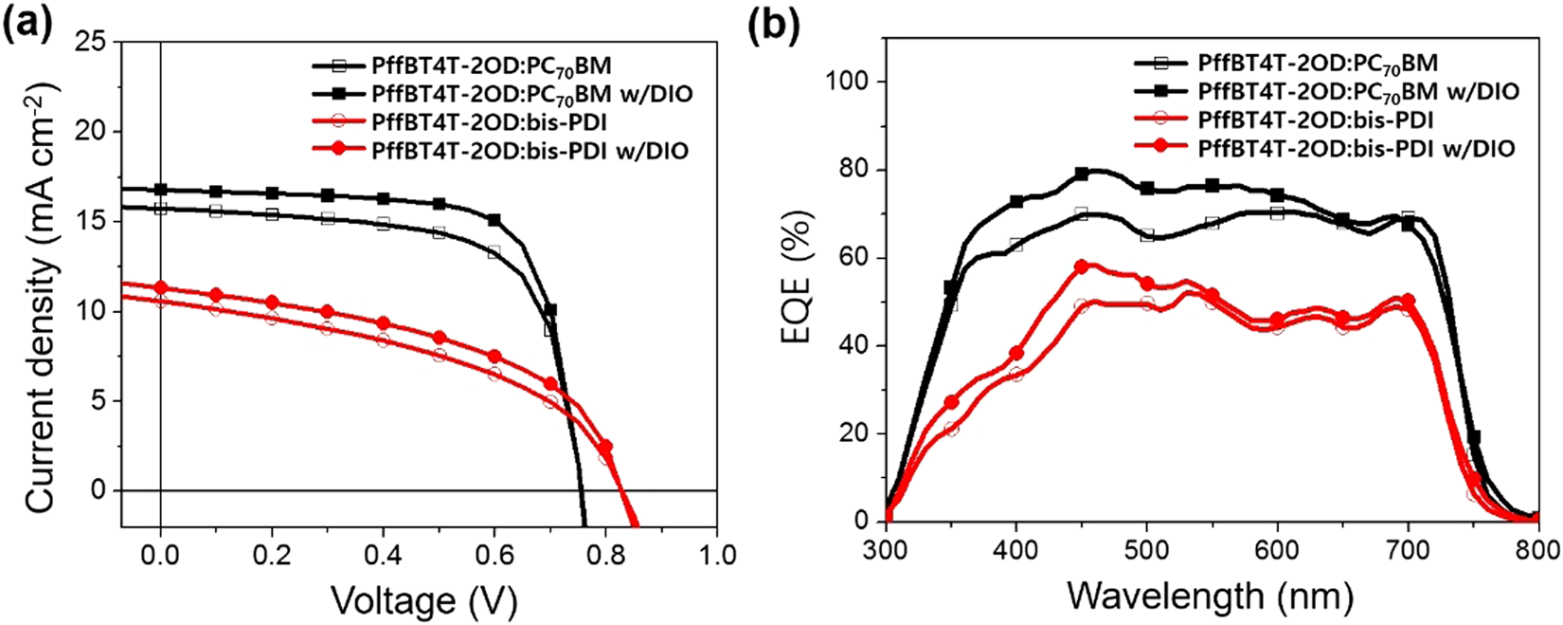
(**a**) *J*-*V* curves and (**b**) EQE spectra of PffBT4T-2OD:PC_70_BM and PffBT4T-2OD:bis-PDI based BHJ OSCs.

BHJ OSCs were fabricated with device structure of glass/indium tin oxide (ITO)/zinc oxide (ZnO)/donor:acceptor/vanadium oxide (V_2_O_5_)/Ag, and current density-voltage (*J*-*V*) characteristics of the optimized PffBT4T-2OD:PC_70_BM and PffBT4T-2OD:bis-PDI OSCs are shown in Fig. 2a. The optimal device performance of the PffBT4T-2OD:bis-PDI system was achieved using a 1:1.5 (wt:wt) ratio and of the PffBT4T-2OD:PC_70_BM system using a 1:1.2 (wt:wt) ratio (Table 1 and S1)^25^. The PffBT4T-2OD:PC_70_BM OSCs exhibited a maximum PCE of 10.19%, with a *V*_OC_ of 0.76 V, a short-circuit current density (*J*_SC_) of 16.82 mA cm^−2^, and a fill factor (FF) of 76.4%, whereas the PffBT4T-2OD:bis-PDI solar cell showed a PCE of 5.18%, a *V*_OC_ of 0.85 V, a *J*_SC_ of 11.22 mA cm^−2^, and a FF of 52.6% with DIO (w/DIO). The high value of V_OC_ for PffBT4T-2OD:bis-PDI OSC is assigned to the high LUMO energy level of the bis-PDI acceptor^26^.

**Table 1:** Photovoltaic properties of the PffBT4T-2OD:PC_70_BM, PffBT4T-2OD:bis-PDI OSCs under standard AM1.5 G illumination. The averages are from over eight devices.

System	*V*_OC_(V)	*J*_SC_ (mA cm^−2^)	*FF* (%)	PCE_ave_ (%)	PCE_max_ (%)	*μ*_e_ (cm^2^ V^−1^ s^−1^)	*μ*_h_(cm^2^ V^−1^ s^−1^)
PffBT4T-2OD:PC_70_BM	0.76 ± 0.002	15.69 ± 0.14	69.3 ± 0.83	8.18 ± 0.13	8.31	6.4 × 10^−4^	1.09 × 10^−4^
PffBT4T-2OD:PC_70_BM w/DIO	0.76 ± 0.001	16.82 ± 0.27	76.4 ± 1.27	9.94 ± 0.25	10.19	1.2 × 10^−3^	4.39 × 10^−4^
PffBT4T-2OD:bis-PDI	0.85 ± 0.004	10.56 ± 0.09	48.9 ± 1.08	4.37 ± 0.16	4.53	2.52 × 10^−6^	3.04 × 10^−4^
PffBT4T-2OD:bis-PDI w/DIO	0.85 ± 0.003	11.22 ± 0.12	52.6 ± 1.15	5.02 ± 0.16	5.18	4.86 × 10^−6^	5.67 × 10^−4^

The external quantum efficiency (EQE) spectra of the PffBT4T-2OD:PC_70_BM and PffBT4T-2OD:bis-PDI devices were recorded over a broad range 300–800 nm (Fig. 2b), which is corresponding well to the absorption spectra of the respective films (Fig. 1c and Figure S2a). The maximum EQE values of the PffBT4T-2OD:bis-PDI (50.1%) and PffBT4T-2OD:bis-PDI w/DIO (58.3%) devices are significantly lower than those of the PffBT4T-2OD:PC_70_BM (69.8%) and PffBT4T-2OD:PC_70_BM w/DIO (79.6%) devices, measured at 455 nm. The large difference in EQE values between bis-PDI- and PC_70_BM-based devices is well matched to the *J*_SC_ values. The *J*_SC_ values calculated from the EQE spectra are in a good agreement with experimental *J*_SC_ in the direct *J*-*V* measurements (Table S2).

The OSC devices were further set to characterize the effective exciton dissociation into free charge carriers in bis-PDI- and PC_70_BM-based devices. For this, we have studied the relationship between the photocurrent (*J*_ph_) and the effective voltage (*V*_eff_) (Figure S2b), *J*_ph_ = *J*_L_ − *J*_D_, where *J*_L_ is the current density under illumination and *J*_D_ is the current density under dark conditions^27^. At a high reverse biased voltage, *J*_ph_ values are saturated in all solar cells. The saturation current density (*J*_sat_) and charge dissociation probability (*P*_diss_ = *J*_ph_/*J*_sat_) were determined for all devices. The calculated *P*_diss_ values under short-circuit condition are 84.6% for PffBT4T-2OD:PC_70_BM, 73.6% for PffBT4T-2OD:bis-PDI, 91.8% for PffBT4T-2OD:PC_70_BM w/DIO, and 74.1% for PffBT4T-2OD:bis-PDI w/DIO, respectively. The higher values of *P*_diss_ obtained from the PffBT4T-2OD:PC_70_BM compared to PffBT4T-2OD:bis-PDI suggest an efficient dissociation of excitons into free charge carriers and better collection of free charge carriers at electrodes^27^.

### Film morphology and photoluminescence quenching

**Figure 3:**
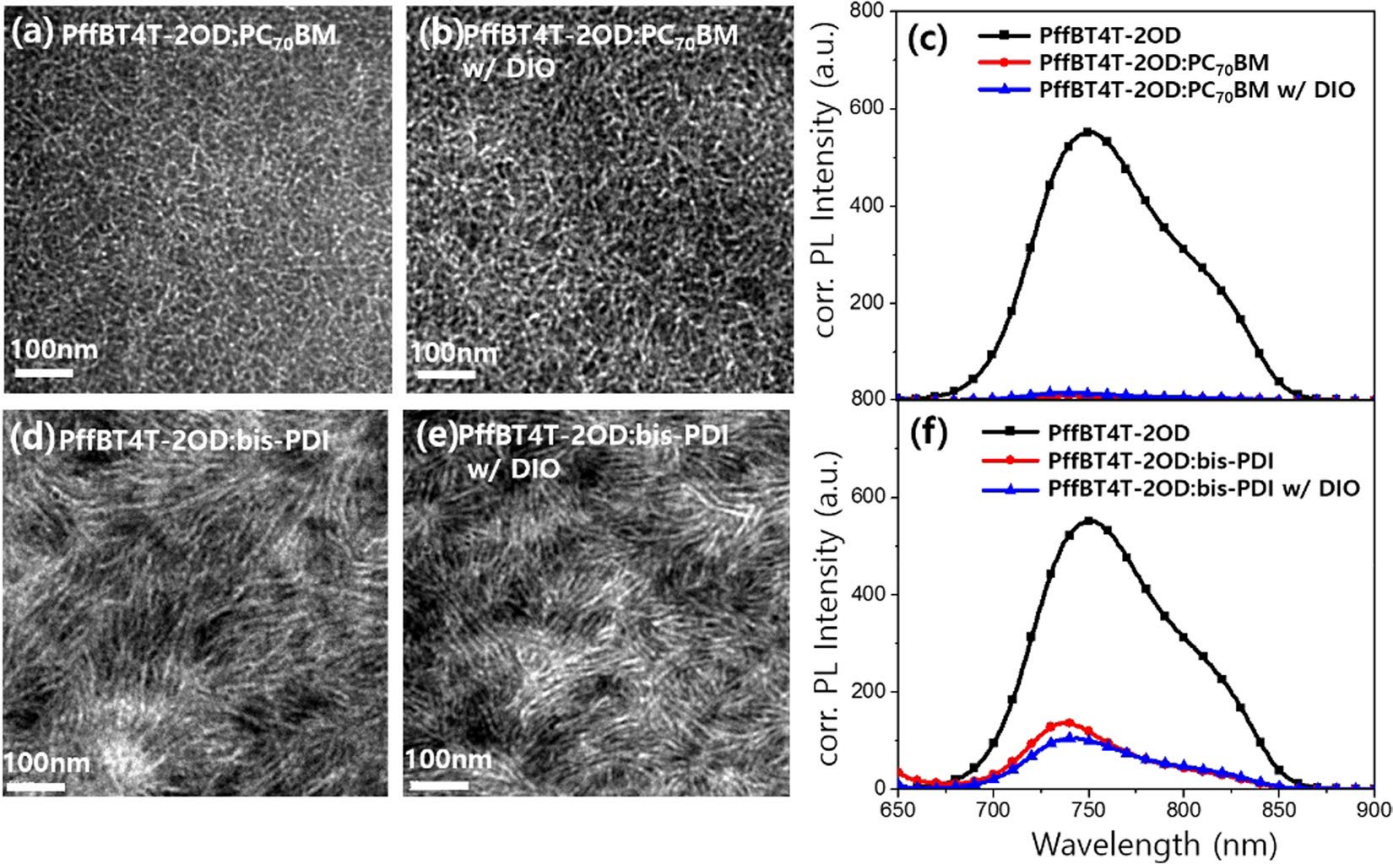
TEM images of (**a**) PffBT4T-2OD:PC_70_BM and (**b**) PffBT4T-2OD:PC_70_BM w/DIO, and (**d**) PffBT4T-2OD:bis-PDI and (**e**) PffBT4T-2OD:bis-PDI w/DIO. Figure (**c**) and (**f**) are the corresponding PL quenching spectra for the blend films with respect to PffBT4T-2OD polymer film, where all films were excited at 632 nm wavelength and PL spectra corrected for the absorption of the films at the wavelength of excitation.

The morphologies of the PffBT4T-2OD:PC_70_BM and PffBT4T-2OD:bis-PDI blend films were characterized by HRTEM (Fig. 3a,b,d,e). All the TEM images of PffBT4T-2OD:PC_70_BM and PffBT4T-2OD:bis-PDI films have showed a nanofibrillar structures kind of BHJ morphology. These fibrils arise from the highly crystalline features of the PffBT4T-2OD polymer^28^, which was also confirmed in GIWAXS experiments (discussed later in this section). In Fig. 3, TEM images depicted that components in PffBT4T-2OD:bis-PDI film are closely packed, and are contained longer fibrils when compare to the PffBT4T-2OD:PC_70_BM blend film where fibrils appear smaller and farther apart. We assume that these differences arose due to the distinct structure and aggregation properties of PC_70_BM and bis-PDI molecules. The slightly coarser morphology in PffBT4T-2OD:bis-PDI blend film reflects in a smaller PL quenching efficiency of polymer PL spectrum (Fig. 3c,f)^29^. The higher value of PL quenching for PffBT4T-2OD:PC_70_BM (93.2%) indicates a better exciton dissociation at the donor/acceptor interface than in the PffBT4T-2OD:bis-PDI (79.9%). The addition of the DIO additive further improved the PL quenching efficiency significantly to 95.6% for PffBT4T-2OD:PC_70_BM and 83.1% for PffBT4T-2OD:bis-PDI due to the effect of DIO on the organization of the PffBT4T-2OD polymer^30^. We have also investigated the PL quenching efficiency of bis-PDI (excited at 532 nm), revealing almost complete quenching in the PffBT4T-2OD:bis-PDI blend films (Figure S3). The complete quenching of bis-PDI excimers implies that bis-PDI is relatively close to the polymer or bis-PDI domain sizes lying well within the excimer diffusion length, so bis-PDI excimers could reach a heterojunction before emitting^31,32^. The AFM images of these two systems are showed in Figure S4. The surface roughness values of the PffBT4T-2OD:PC_70_BM blend system decreased w/DIO treatment from 1.8 nm to 1.1 nm, whereas surface roughness of the PffBT4T-2OD: bis-PDI film increased from 1.9 nm to 2.8 nm.

The differences in blend morphologies of two systems might have arisen due to the different surface energies of the components. Therefore, the surface energies of the pristine films were evaluated by measuring the contact angles formed between the film and either of two different liquids (deionized (DI) water and ethylene glycol (EG)) (Figure S5). Later, contact angles were used in the Owens and Wendt geometric mean equation to calculate the surface energies (Table S3)^33^. The relatively large difference in surface energies of the PffBT4T-2OD (*γ* = 29.1 mN m^−1^) and the bis-PDI (*γ* = 41.1 mN m^−1^) is likely to result in a poor miscibility in the blend film, producing a slightly larger phase-separated film morphology. Whereas in the PffBT4T-2OD:PC_70_BM blend film, the spherical shape fullerene molecule and small difference in surface energies of the components enabled the PC_70_BM (*γ* = 31.6 mN m^−1^) molecule to easily penetrate and diffuse into the polymer region, which may have hindered strong aggregation of polymer^34,35^. However, the bis-PDI molecule could not penetrate into the polymer region because of large surface energy difference, and allow polymer to crystallize largely, as can be seen in TEM images. The high surface energy of the bis-PDI film may be because of more nitrogen and oxygen atoms in molecular structure compared to PC_70_BM^36^.

**Figure 4:**
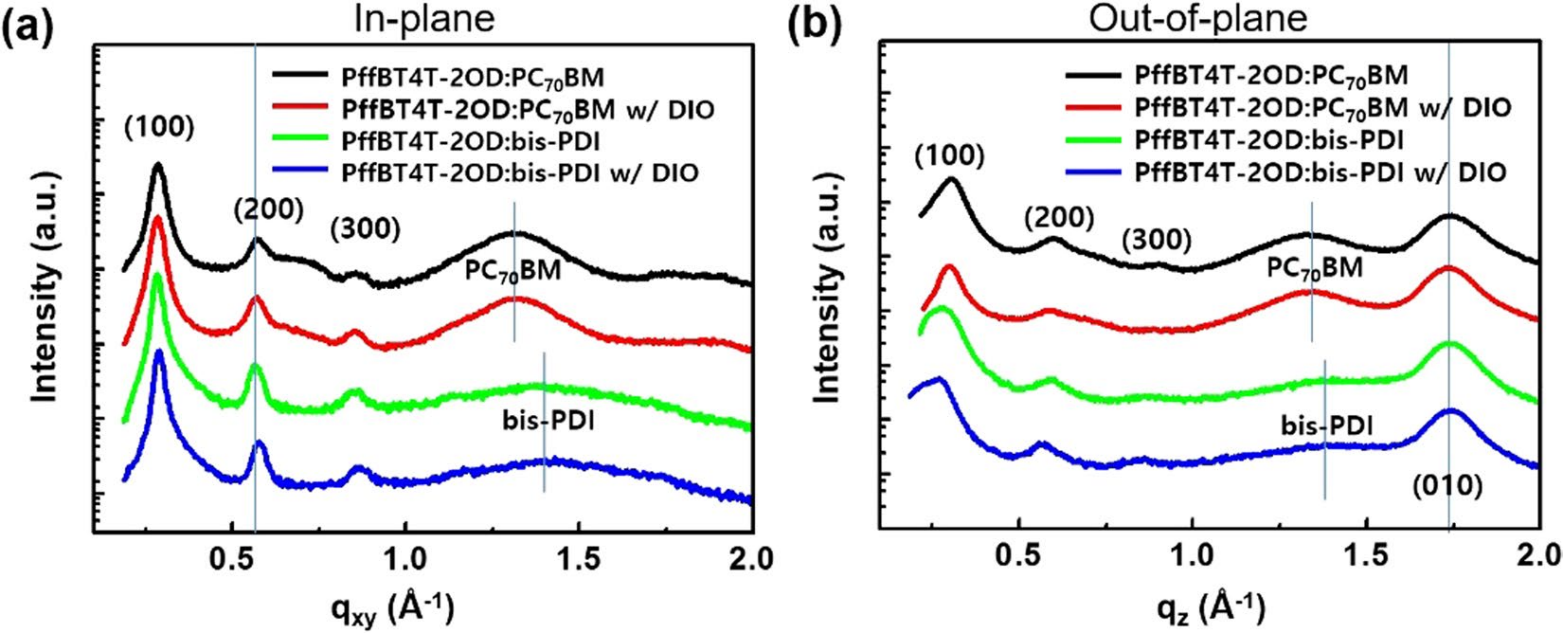
(**a**) In-plane and (**b**) out-of-plane scan for the blend films of PffBT4T-2OD:PC_70_BM, PffBT4T-2OD:PC_70_BM w/DIO, PffBT4T-2OD:bis-PDI and PffBT4T-2OD:bis-PDI w/DIO.

The crystalline properties of the PC_70_BM and bis-PDI acceptors in the presence of the PffBT4T-2OD polymer were investigated using GIWAXS experiments (Figures S6, S7). The information extracted from the in-plane and out-of-plane of GIWAXS images are presented in Fig. 4 and summarized in Table S4. In the 2D-XRD images, both blend films exhibit distinctive diffraction peaks, as evidenced by the strong lamellar stacking with (100), (200), and (300) diffraction peaks, as well as the distinct (010) diffraction peak present in the out-of-plane direction, which indicated the presence of a preferential face-on orientation of PffBT4T-2OD polymer domains. In Figure S6a, three isotropic diffraction rings at *q* = 0.63 Å^−1^, 1.28 Å^−1^, and 1.90 Å^−1^ represent the amorphous behavior of the PC_70_BM with randomly oriented molecules^37^. On the other hand in Figure S6b, the pristine bis-PDI film showed a stronger (100) diffraction peak in the out-of-plane profile and a peak near 1.5 Å^−1^, which corresponds to the *π*-*π* stacking interactions, is more distinctive in the in-plane direction. These features of the in-plane and out-of-plane profiles of the bis-PDI film suggested that the preferred molecular orientation was weakly edge-on. In Figures S7a,c, the broad peaks at *q* = 1.3 Å^−1^ are the characteristic of PC_70_BM aggregation^38^ and a weak peak near *q* = 1.42 Å^−1^ represents bis-PDI aggregation. To understand the impact of two structurally different small molecules (bis-PDI and PC_70_BM) on the polymer chain packing, we have analyzed the coherence length for polymer peak (200) in both the blend systems. The coherence lengths for the (200) diffraction peak in the in-plane direction and the (010) diffraction peak in the out-of-plane direction were calculated for all blend systems using the Scherrer equation^39^. The coherence length in the PffBT4T-2OD:bis-PDI (15.6 nm) was much larger than that in the PffBT4T-2OD:PC_70_PM (11.8 nm), suggesting that the PffBT4T-2OD polymer formed larger crystals when mixed with bis-PDI than with PC_70_BM. Moreover, the PffBT4T-2OD:bis-PDI films prepared with DIO formed larger *π*-*π* stacked crystals (5.0 nm) oriented in the out-of-plane direction than were formed in the PffBT4T-2OD:PC_70_PM blend film (4.3 nm). Here, we speculate that the planar molecular structure of bis-PDI would form *π*-*π* interactions with PffBT4T-2OD, thereby favoring the crystallization of polymer molecules with a face-on orientation, in contrast with the PffBT4T-2OD:PC_70_BM blend film.

### Charge transport properties

The electron and hole mobilities of the two blend systems were evaluated using the space charge limited current (SCLC) method^18^ with controlled device structures: ITO/ZnO/donor:acceptor/Ca/Al for electron-only and ITO/PEDOT:PSS/donor:acceptor/Au for hole-only devices (Table 1, Figure S8)^18^. The PffBT4T-2OD:PC_70_BM device has shown a better electron mobility (*μ*_e_ = 6.4 × 10^−4^ cm^2^ V^−1^s^−1^) as compared to the PffBT4T-2OD:bis-PDI system (*μ*_e_ = 2.52 × 10^−6^ cm^2^ V^−1^s^−1^), whereas the hole mobility of the PffBT4T-2OD:bis-PDI system (*μ*_h_ = 3.04 × 10^−4^ cm^2^ V^−1^s^−1^) is 2.8 times higher to the PffBT4T-2OD:PC_70_BM system (*μ*_h_ = 1.09 × 10^−4^ cm^2^ V^−1^s^−1^). The ratio of *μ*_e_/*μ*_h_ for the PffBT4T-2OD:PC_70_BM is much closer to 1, indicating good balance of charge transport in the devices. The balanced and high mobility values obtained from PffBT4T-2OD:PC_70_BM explained, in part, the higher values of *FF* and PCE obtained from OSC devices.

### Charge carrier recombination dynamics

**Figure 5:**
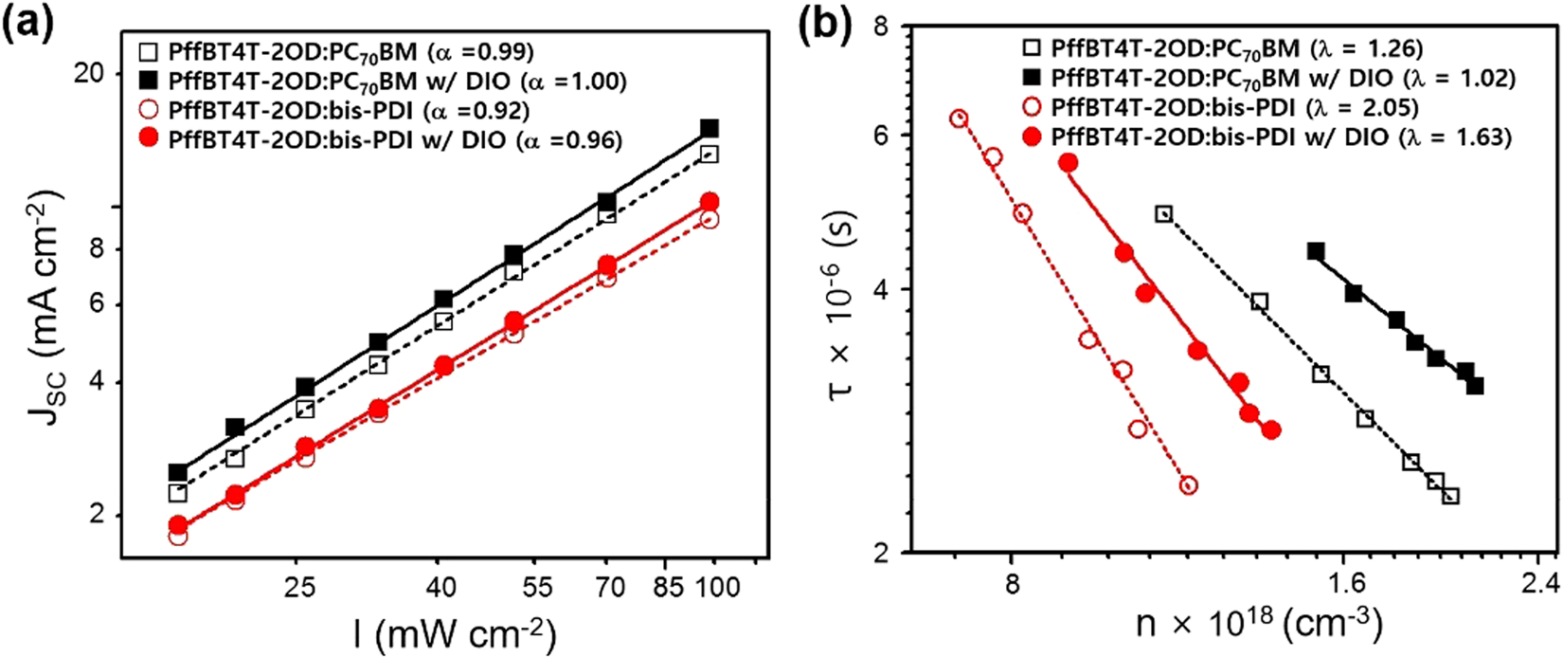
(**a**) Light intensity (*I*) dependence current density (*J*_SC_) and (**b**) charge carrier lifetime (τ) versus charge carrier density (n) for PffBT4T-2OD:PC_70_BM and PffBT4T-2OD:bis-PDI based OSCs. Scattered points represent experimental data points, while the dashed and solid lines demonstrate the fitting according to the equations ${J}_{SC}\propto {I}^{\alpha }$ in (**a**) and $\tau {(}{n}{)}{=}{\tau }_{{0}}{{n}}^{{-}\lambda }$ in (**b**), respectively^41,48^.

In order to gain insight into the charge carrier recombination dynamics in both type of OSCs, we varied light intensity during the measurement of *J*-*V* characteristics and the results are shown in Fig. 5a. A plot of *J*_SC_ versus the light intensity (*I*) on a log-log scale provides a good comparison of the non-geminated bimolecular recombination losses at 0 V, where *I* was varied over the range 17–100 mW cm^−2 ^^40^. The experimental data points showed in Fig. 5a were fitted to a power law^41^ ${J}_{SC}\propto {I}^{\alpha }$ as a linear fit on the logarithmic scale. The linear fits provided the exponents *α* = 0.99 for the PffBT4T-2OD:PC_70_BM, *α* = 1.00 for the PffBT4T-2OD:PC_70_BM w/DIO, *α* = 0.92 for the PffBT4T-2OD:bis-PDI and *α* = 0.96 for the PffBT4T-2OD:bis-PDI w/DIO systems. A stronger deviation of *α* from 1 for the bis-PDI-based OSCs indicates that *J*_SC_ for these devices is limited by bimolecular recombination losses^40^, however such deviation in *α* could also be assign to space charge limited effects^42,43^ or to inefficient charge extraction caused by an improper choice of charge carrier-collecting electrodes^44,45^.

The difference in charge carrier recombination dynamics of the fullerene and bis-PDI-based OSCs is quantified by measuring the TPV and transient photocurrent (TPC). The charge carrier lifetime ($\tau$) and carrier density (*n*) were estimated from the TPV and TPC data as described earlier^46,47^. The carrier density versus carrier lifetime is plotted in Fig. 5b and fitted to a power law equation $\tau (n)={\tau }_{0}{n}^{-\lambda }$, where τ_0_ is the intercept at *n* = 0, and *λ* is the magnitude of the slope, which is related to the order of the non-geminated recombination (*λ* + 1)^48^. For similar values of *n* = 1.15 × 10^18^ cm^−3^, the charge carrier lifetime in PffBT4T-2OD:PC_70_BM is double to that in the PffBT4T-2OD:bis-PDI device, indicating a much slower recombination rate in the fullerene-based OSCs. Further, the carrier lifetime of the devices increased with the use of the additive, consistent with the improved device performance. The slope *λ* extracted 1.26 for PffBT4T-2OD:PC_70_BM, 1.02 for PffBT4T-2OD:PC_70_BM w/DIO, 2.05 for PffBT4T-2OD:bis-PDI, and 1.63 for PffBT4T-2OD:bis-PDI w/DIO suggesting that the bis-PDI-based devices severely affected by the non-geminated trap-assisted recombination, whereas the fullerene-based devices are nearly independent of trap-assisted recombination^48^. We expect unbalanced charge carrier transport and largely phase-separated morphology in PffBT4T-2OD:bis-PDI system are the main reasons for having high trap-assisted recombination. To understand the role of the charge carrier transport and the morphology of the blend systems on recombination dynamics, we have performed temperature-dependent SCLC mobility and *J*-*V* measurements under different sunlight intensities.

### Temperature-dependent measurements

**Figure 6:**
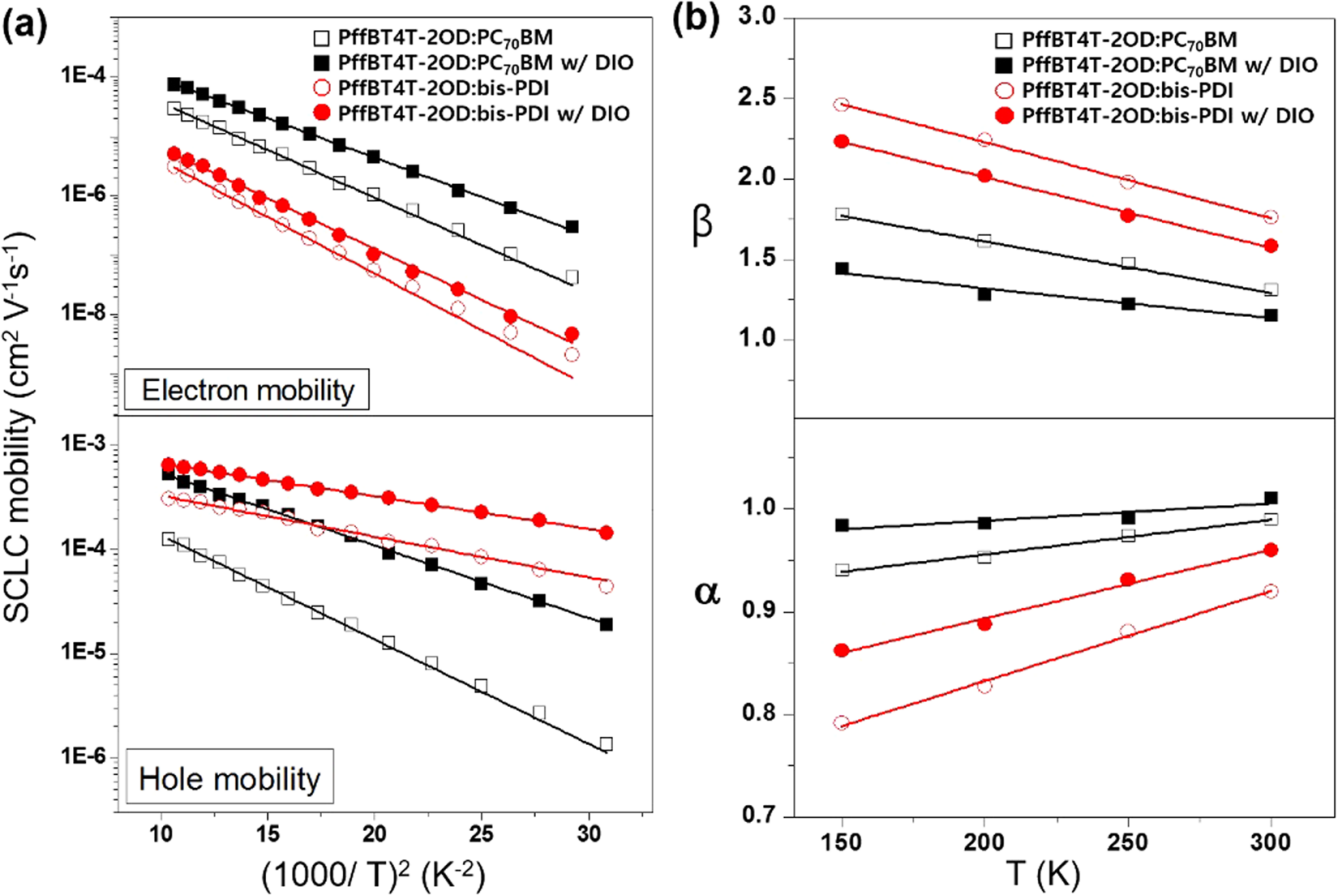
(**a**) Electron and hole mobility versus 1/T^2^ plot fitted according to the Gaussian disorder model^50^ (**b**) α and β parameters plotted with different temperatures for PffBT4T-2OD:PC_70_BM and PffBT4T-2OD:bis-PDI based devices. In Figure (**b**), α and β parameters are extracted from temperature- and intensity-dependent *J*-*V* characteristics in supporting information (Figures S9, S10) and the solid line is a guideline for the eyes.

Temperature-dependent SCLC charge carrier mobilities were determined from dark *J*-*V* characteristics measured at various temperatures between 150–310 K. Figure 6a shows the electron and hole mobility values of the PffBT4T-2OD:PC_70_BM and PffBT4T-2OD:bis-PDI systems as a function of the inverse square of the temperature, *T*^−2^. The curves show a fit to the equation $\mu ={\mu }_{\infty }{e}^{{(-{T}_{0}/T)}^{2}}$, where ${\mu }_{\infty }$ is the mobility at infinite temperature and *T*_0_ is the characteristic temperature^49^. The slope of the fitted line is related to the width of the density of states through equation $\sigma =\frac{3}{2}{k}_{B}{T}_{0}$, where *k*_*B*_ indicates the Boltzmann constant and derived values of σ are listed in Table S5. The energetic disorder parameter *σ*_e_ for electron transport in PffBT4T-2OD:PC_70_BM (74.6 meV) is smaller than that in PffBT4T-2OD:bis-PDI (81.5 meV), supporting a higher electron mobility in the PffBT4T-2OD:PC_70_BM blend system. Also, the large difference in *σ*_h_ values for PffBT4T-2OD:PC_70_BM (62.0 meV) and PffBT4T-2OD:bis-PDI (38.8 meV) explains the difference in hole mobility values of the two systems. It seems evident that the values of *σ*_h_ strongly depends on the PffBT4T-2OD polymer domains in two blend systems. The large difference in device performance might arise from the differences in the energetic disorder and, as a result, slower charge carriers start accumulating in the photoactive layer and increase the recombination losses^50^.

To understand the effect of energetic disorder directly on the non-geminated bimolecular and trap-assisted recombinations, we measured intensity-dependent *J*_SC_ and *V*_OC_ at various low temperatures 150 K, 200 K, 250 K and 300 K (Figures S9 and S10). In supporting information, the intensity-dependent *J*_SC_ data was fitted with power law and the α values were extracted in similar method as described above. The exponent α decreased from 0.99 to 0.94 (PffBT4T-2OD:PC_70_BM), 1 to 0.98 (PffBT4T-2OD:PC_70_BM w/DIO), 0.91 to 0.79 (PffBT4T-2OD:bis-PDI), and 0.96 to 0.86 (PffBT4T-2OD:bis-PDI w/DIO) as the measuring temperature decreased (Fig. 6b). The decrease in α could be explained in terms of slow charge extraction at a reduced mobility, which increase the availability of free charge carriers to recombine^50^. The faster rate of α decay with decreasing temperature suggests a higher-order bimolecular recombination losses in the PffBT4T-2OD:bis-PDI system. The intensity-dependent *V*_OC_ was fitted to equation *V*_OC_ ∝ *β*(*k*_B_*T*/*e*)ln(*I*)^41^, where T is the absolute temperature, and e is the electron charge (Figure S10). The extent of trap assisted recombination can be estimated by extracting the *β* values^40^. *β* = 1 implies no trap-assisted recombination, whereas *β* > 1 indicates that trap-assisted recombination is dominating mechanism. The higher values of *β*, and the faster rate of increase in *β* values with decreasing temperature obtained for the PffBT4T-2OD:bis-PDI devices suggest that the devices are strongly limited by the trap-assisted recombination in energetically localized states. The increase in *β* with decreasing temperature is expected because of the stronger occupation of deeply localized states (traps), as the charge carriers no longer had access to the thermal energy needed to escape from the trap states^51^.

## Conclusions

In conclusion, we have performed a comprehensive comparative analysis for elucidating the key morphological issues that are responsible for low performance of bis-PDI-based OSCs. We observed that the morphological differences in blend films, that determined the device performance, are arisen due to the distinct miscibility of PC_70_BM and bis-PDI molecules with PffBT4T-2OD polymer. GIWAX, TEM and AFM studies revealed the strongly face-on oriented PffBT4T-2OD polymer forms a well-mixed interconnected BHJ morphology with amorphous PC_70_BM instead of weakly edge-on oriented bis-PDI molecule. In addition, the large coherence length in the PffBT4T-2OD:bis-PDI suggested that bis-PDI molecule favoring the crystallization of PffBT4T-2OD polymer molecules. However, the lower values of *J*_SC_ and *FF* in PffBT4T-2OD:bis-PDI are primarily attributed to the weak absorption in near-UV region, inefficient charge generation, low electron mobility and unbalanced charge carrier transport. The relevance of unbalanced charge transports in PffBT4T-2OD:bis-PDI devices was supported by their large differences in the energetic disorder associated with electron and hole transports. Further, low-temperature light-intensity dependent study on devices confirmed the dominance of trap-assisted recombination in PffBT4T-2OD:bis-PDI devices.

## Methods

### Materials

PffBT4T-2OD and PC_70_BM were purchased from Solarmer Energy Inc., and were used as received. The bis-PDI small molecule was synthesized according to the published procedure^52^. A ZnO precursor solution was prepared by dissolving 1 g zinc acetate dihydrate (99.999% trace metals basis, Aldrich) in 10 mL 2-methoxyethanol (99.8%, anhydrous, Aldrich) with 0.28 g ethanolamine (≥99.5%, Aldrich) as a surfactant, followed by stirring overnight under ambient conditions^53^.

### Characterization of the Materials

The bis-PDI and other intermediate were characterized by ^1^H NMR, ^13^C NMR, and mass spectrometry (MALDI-TOF). The LUMO levels of bis-PDI and PC_70_BM were calculated from cyclic voltammetry measurements. The UV-Vis absorption and PL spectra of the spin-coated films were recorded using a Perkin Elmer, Lambda 1050 spectrometer with a Horiba Jobin Yvon NanoLog spectrofluorimeter, respectively.

### Preparation of the solar cells

The inverted structure of the OSC devices was prepared with the structure: stack glass/ITO (110 nm)/ZnO (40 nm)/donor:acceptor/V_2_O_5_ (2 nm)/Ag (100 nm). ITO-coated glass substrates were cleaned by sequential sonications in detergent, distilled water, acetone, and isopropyl alcohol for 15 minutes at each step. After UV/ozone treatment for 30 minutes, a ZnO electron transport layer^7^ was prepared by spin-coating at 4000 rpm and then baking at 120 °C for 30 minutes on the hot plate under ambient conditions. The photoactive layer solutions were prepared in CB (polymer concentration: 9 mg mL^−1^) and were heated on a hotplate at 110 °C for 8 hours with stirring. The photoactive layers were spin-coated from the warm solution onto the prepared glass/ITO/ZnO substrate in a N_2_-filled glovebox. The films were then annealed at 100 °C for 5 minutes. The electrodes were deposited by transferring the samples into a vacuum chamber. V_2_O_5_ (2 nm)/Ag (100 nm) was then thermally deposited on top of the photoactive layer with the help of a shadow mask having a 0.0555 cm^2^ device area.

### Characterization of the solar cells

The electrical characteristics were measured using a Keithley 4200 unit under 100 mW cm^−2^ AM1.5 solar illumination in a N_2_-filled glove box. The light was generated using an Oriel 1-kW solar simulator referenced using a Reference Cell PVM 132 calibrated at the US National Renewable Energy Laboratory. The external quantum efficiency was measured using a photomodulation spectroscopy setup (Merlin, Oriel) with monochromatic light from a Xenon lamp. The power density of the monochromatic light was calibrated using a Si photodiode certified by the National Institute for Standards and Technology. Light intensity-dependent *J*-*V* curves were measured using the same solar simulator setup by varying the intensity from 0.17–1 sun.

### Preparation of the control devices and their characterization

The hole- and electron-only control devices were prepared with the device structure ITO/PEDOT:PSS/donor:acceptor/Au and ITO/ZnO/donor:acceptor/Ca/Al, respectively. The electrical characteristics were measured using the Keithley 4200 in a N_2_-filled glove box. The mobility values were extracted from the dark *J*-*V* characteristics of the devices by fitting to the Mott-Gurney equation^18^.

### Morphological characterization

AFM and TEM images are obtained using a MultiMode 8 Scanning Probe Microscope from VEECO Instruments Inc. and a JEOL JEM-2200FS (with an Image Cs-corrector, bright field mode), respectively. TEM samples were prepared on water soluble sacrificial substrates (glass/ITO/PEDOT:PSS) and floated onto the surface of DI water before being transferred on 200 mesh copper grids for top-down imaging. GIWAXS analysis was performed using Beamline 3 C at the Pohang Accelerator Laboratory (PAL). The photon energy was 10.6408 keV (λ = 1.1651 Å). The beam size was adjusted to 0.8 mm × 0.8 mm in width and height, respectively. Rayonix MAR165 detector was used for data collection. The thin film samples were prepared on silicon wafer and deposited in the same way as when making the solar cell devices. The angle between the film surface and the incident beam was fixed at 0.12° for all of the samples. The GIWAXS images shown are normalized with respect to exposure time. The contact angle measurements were carried out under atmospheric conditions using SEO330A (Surface and Electro-Optics).

### Charge carrier dynamics

TPV and TPC were measured using a TDC3054C digital oscilloscope connected to high-speed reamplifiers: SR560 and DHPCA-100. The samples were excited by a 3 ns pulsed laser at 532 nm (OBB, NL4300, and OD401) under AM1.5 G illumination at an intensity of 0.3–1 sun.

### Low-temperature measurements

Low-temperature measurements were conducted by supporting the devices using a standard sample holder in a liquid N_2_ cryostat (JANIS VPF-100). The low temperatures (150–300 K) of the devices were controlled by connecting the cryostat to a Lake Shore, 335 temperature controller unit, and dark *J*-*V* characteristics were measured using a Keithley 2636 source meter.

## Electronic supplementary material

Supplementary Information
