# Supplementary material for: Unraveling the efficiency-limiting morphological issues of the perylene diimide-based non-fullerene organic solar cells
Source: Sci Rep. 2018 Feb 12;8:2849. doi: 10.1038/s41598-018-21162-x (PMC5809366; doi:10.1038/s41598-018-21162-x)
Supplement: Supplementary file 1 — Supplementary Information [file 41598_2018_21162_MOESM1_ESM.pdf]

Supporting information

Unraveling the efficiency-limiting morphological issues of the perylene diimide-based non-fullerene organic solar cells

Ranbir Singh, Sanjaykumar R. Suranagi, Jaewon, Lee, Hansol Lee, Min Kim, Kilwon Cho*

Department of Chemical Engineering, Pohang University of Science and Technology, Pohang, 37673, Korea

*Corresponding to kwcho@postech.ac.kr

Experimental section.

Materials and Methods. All reagents and chemicals were purchased from commercially available sources and used without further purification. Anhydrous organic solvents for the synthesis and characterization, including chloroform and DMSO were purchased from Sigma-Aldrich. Perylene-3,4,9,10-tetracarboxylic dianhydride and imidazole were purchased from TCI. Copper nanoparticles (60 ~ 80 nm) was purchased from SkySpring Nanomaterials, Inc. All chlorinated solvents were purchased from Sigma-Aldrich, Inc. 1-Hexylheptylamine,[1] N,N'-bis(1-hexylheptyl)perylene-3,4,9,10-tetracarboxylbisimide (PDI1),[1] 1Br-PDI1,[2] and bis-PDI[3] were synthesized using modified literature procedure.

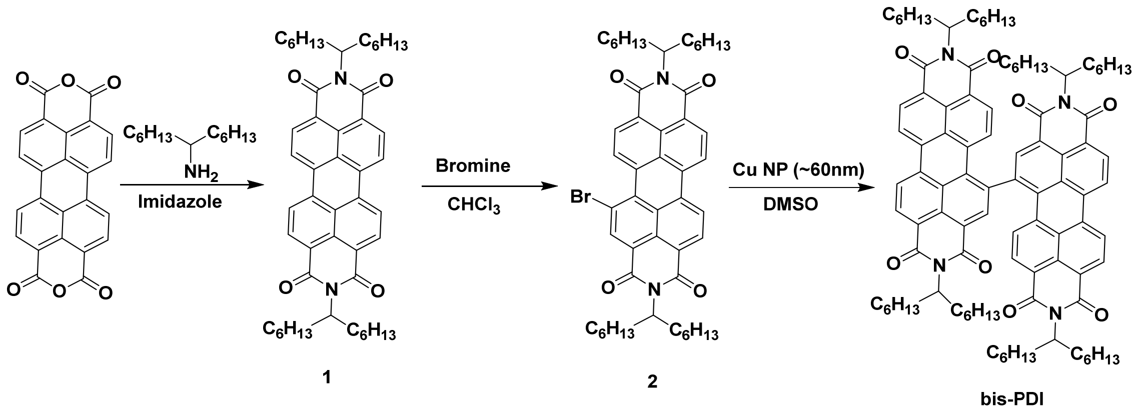

Scheme S1: Synthetic route for bis-PDI

*Synthesis of N,N'-bis(1-hexylheptyl)perylene-3,4,9,10-tetracarboxylicdimide (**1**).*

A mixture of perylene-3,4,9,10-tetracarboxylicdianhydride (3.9 g, 9.94 mmol), 1-hexylheptylamine (5.05 g, 25.34 mmol), and imidazole (40 g) were stirred at 120°C for 24 h. The reaction mixture was cooled to room temperature and taken up in 150 mL ethanol, treated with 200 mL 2 M HCl, and stirred for 6 h. The dark red precipitate was filtered and washed with distilled water followed by methanol. After drying in vacuum oven the crude compound was subjected to flash column chromatography purification, using silica gel (*n*-hexane:chloroform = 1 : 3) to afford the product **1** as dark red solid (6.6 g, 88%). **^{1}H NMR** (600.27 MHz, $CDCl_3$) (δ ppm): 8.66 ~ 8.58 (m, 4H), 8.57 (d, *J*=7.92 Hz, 4H), 5.15 ~ 5.05 (m, 4H), 2.22 ~ 2.13 (m, 4H), 1.83 ~ 1.75 (m, 4H), 1.31 ~ 1.11 (m, 32H), 0.75 (t, *J*=6.9 Hz, 12H); **^{13}C NMR** (150.938 MHz, $CDCl_3$) (δ ppm): 164.59, 163.51, 134.40, 131.80, 131.06, 129.53, 126.36, 123.92, 123.19, 122.93, 54.77, 32.38, 31.76, 29.22, 26.95, 22.59, 14.03. Calculated mass for $C_{50}H_{62}N_2O_4$: 755.06, **MALDI-TOF** Mass obtained: 756.5.

*Synthesis of 1-bromo-N,N'-bis(1-hexylheptyl)perylene-3,4,9,10-tetracarboxylicdimide (**2**).*

To a 3-neck round flask charged with a solution of compound **1** (5.1 g, 6.7 mmol) in $CHCl_3$ (100 mL) was added bromine (12 mL) and stirred at room temperature for 3 days. After cooling to 0 - 5°C the reaction mixture was slowly dropped into a cold saturated solution of sodium thiosulphate under stirring. The organic layer separated and washed twice with distilled water (50 mL) and dried over magnesium sulphate. The solvent was evaporated in vacuo and crude product was purified by silica gel flash column chromatography (*n*-hexane:chloroform = 1 : 1 to 1 : 3, v/v) to afford the product **2** as dark red solid (1.38 g, 45%). **^{1}H NMR** (600.27 MHz, $CDCl_3$) (δ ppm): 9.82 (d, *J*=8.4 Hz, 1H), 8.96 (d, *J*=17.4 Hz, 1H, broad), 8.73 (d, *J*=15.0 Hz, 3H, broad), 8.65 ~ 8.63 (m, 2H), 5.24 ~ 5.16 (m, 2H), 2.30 ~ 2.25 (m, 4H), 1.19 ~ 1.85 (m, 4H), 1.38 ~ 1.24 (m, 32H), 0.75 (t, *J*=6.9 Hz, 12H); **^{13}C NMR** (150.938 MHz, $CDCl_3$) (δ ppm): 164.37, 163.56, 163.29, 162.39, 139.58, 138.85, 133.88, 133.52, 131.49, 131.05, 130.14, 128.96, 128.78, 128.18, 127.06, 124.18, 124.01, 120.92, 54.99, 54.80, 32.37, 32.21, 31.76, 29.21, 29.19, 26.92, 26.90, 22.58, 14.03. Calculated mass for $C_{50}H_{61}BrN_2O_4$: 833.95, **MALDI-TOF**

Mass obtained: 834.44.

Synthesis of bis-PDI.

A two neck round bottom flask is charged with **2** (300 mg, 0.36 mmol), copper nanoparticles (60 ~ 80 nm particle size) (230 mg, 3.6 mmol) and dry DMSO (40 ml) under argon. The mixture was heated to 100ºC with vigorous stirring for 12 h. The cooled mixture was poured into water, and the precipitate was collected by vacuum filtration, washed with water, dried, and purified by column chromatography on silica gel, eluted with *n*-hexane/CH_2Cl_2 to afford **bis-PDI** as red-violet solids. **^{1}H NMR** (600.27 MHz, CDCl3) (δ ppm): 8.78 ~ 8.66 (m, 8H), 8.43 (d, *J*=8.1 Hz, 2H), 8.15 ~ 8.05 (m, 4H), 5.10 ~ 4.90 (m, 4H), 2.24 ~ 1.90 (m, 8H), 1.91 ~ 1.57 (m, 8H), 1.30 ~ 1.00 (m, 64H), 0.78 ~ 0.62 (m, 24H); **^{13}C NMR** (150.938 MHz, CDCl3) (δ ppm):164.62, 164.41, 164.35, 163.51, 163.34, 163.18, 163.00, 141.90, 134.83, 134.22, 134.07, 132.90, 132.12, 131.34, 129.62, 128.87, 128.79, 127.49, 127.34, 124.00, 123.40, 54.98, 54.79, 32.63, 32.42, 32.345, 32.32, 32.29, 31.91, 31.88, 31.68, 29.17, 29.11, 27.06, 26.99, 26.83, 22.53, 13.99, 13.97. Calculated mass for $C_{100}H_{122}N_4O_8$: 1508.10, **MALDI-TOF** Mass obtained: 1506.24.

Characterizations of compounds: ^{1}H spectra of all synthesized molecules were recorded on a Bruker Ascend^{TM} 600 MHz spectrometer. Mass spectra were obtained from Bruker Reflex III Matrix-Assisted LASER Desorption Ionization - Time of Flight Mass Spectrometer (MALDI-TOF) using α-Cyano-4-hydroxycinnamic acid (α-CCA) as a matrix[4] recorded in a (+)-reflector mode. Elemental analysis was performed by Vario MICRO.

Electrochemical characterizations: The cyclic voltammetry (CV) was conducted on a Iviumstat. h; Electrochemical Interface & Impedance Analyser instrument with glassy carbon disk, Pt wir e, and Ag/Ag+ electrode as the working electrode, counter electrode, and reference electrode, respectively in a 0.1 M tetrabutylammonium hexafluorophosphate (*n*-Bu4NPF6)-anhydrous acet onitrile solution at a potential scan rate of 50 mV s^{-1}. Thin films of samples were deposited onto the glassy carbon working electrode from a 3.0 mg/mL chlorobenzene solution. The elect rochemical energy levels were estimated by using the empirical formula: E_{HOMO} = - (4.80 +

E_{onset}^{ox} - $Fc_{1/2}$) and E_{LUMO} = - (4.80 + E_{onset}^{red} - $Fc_{1/2}$).[5]

The LUMO level of bis-PDI calculated from above equation is - 3.88 eV

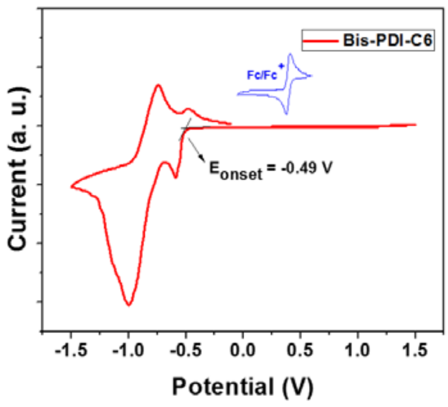

Figure S1. Cyclic voltammogram of bis-PDI.

Table S1. The detailed optimization of photovoltaic performances of the solar cells based on PffBT4T-2OD:bis-PDI with different donor: acceptor (D:A) ratios and DIO vol%.

D:A	V_{OC} (V)	J_{SC} (mA cm^{-2})	*FF* (%)	PCE (%)
PffBT4T-2OD:bis-PDI (1:1)	0.84 ± 0.007	9.47 ± 0.09	46.4 ± 0.67	3.72 ± 0.11
PffBT4T-2OD:bis-PDI (1:1.5)	0.85 ± 0.004	10.56 ± 0.09	48.9 ± 1.08	4.37 ± 0.16
PffBT4T-2OD:bis-PDI (1:2)	0.85 ± 0.003	10.23 ± 0.11	45.8 ± 1.62	3.98 ± 0.13
Additive DIO				
PffBT4T-2OD:bis-PDI + 0.5% DIO	0.85 ± 0.004	10.97 ± 0.17	50.2 ± 1.01	4.69 ± 0.18
PffBT4T-2OD:bis-PDI + 1% DIO	0.85 ± 0.003	11.22 ± 0.12	52.6 ± 1.15	5.02 ± 0.16
PffBT4T-2OD:bis-PDI + 2% DIO	0.85 ± 0.004	10.86 ± 0.15	49.1 ± 0.84	4.53 ± 0.21
PffBT4T-2OD:bis-PDI + 3% DIO	0.85 ± 0.005	10.19 ± 0.08	47.8 ± 0.76	4.14 ± 0.09

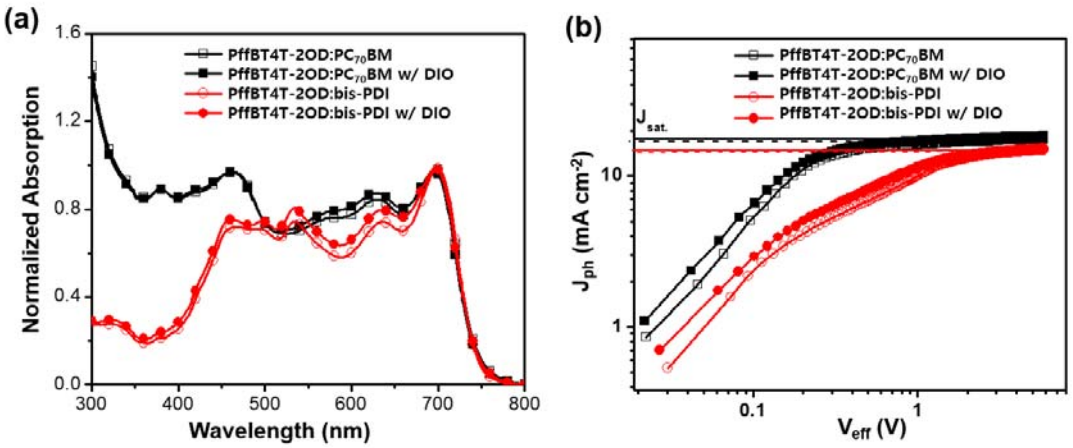

Figure S2. (a) Normalized absorption spectra (b) photocurrent density (J_{ph}) versus effective voltage (V_{eff}) characteristics of PffBT4T-2OD:PC$_{70}$BM and PffBT4T-2OD:bis-PDI (open symbols) and w/DIO additive (solid symbols).

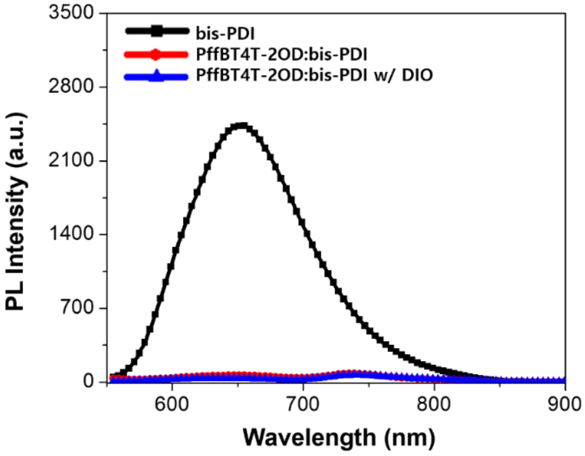

Figure S3. PL spectra of PffBT4T-2OD:bis-PDI and PffBT4T-2OD:bis-PDI w/DIO blend films with respect to bis-PDI film, where all the films were excited at 532 nm wavelength.

Table S2. PL quenching efficiency and $J_{cal.,EQE}$ (calculated from EQE spectrum) for the studied D:A system w/o DIO and w/DIO additive.

D:A	PL quenching efficiency (%) (D)	PL quenching efficiency (%) (A)	$J_{cal., EQE}$ (mA cm^{-2})
PffBT4T-2OD:PC_{70}BM	93.2	---*	15.52
PffBT4T-2OD:PC_{70}BM w/DIO	95.6	---*	16.71
PffBT4T-2OD:bis-PDI	79.9	97.5	10.25
PffBT4T-2OD:bis-PDI w/DIO	83.1	98.7	11.13

*PL from PC_{70}BM is weak and so we cannot extract the PL quenching efficiency for the PffBT4T-2OD:PC_{70}BM blend film.

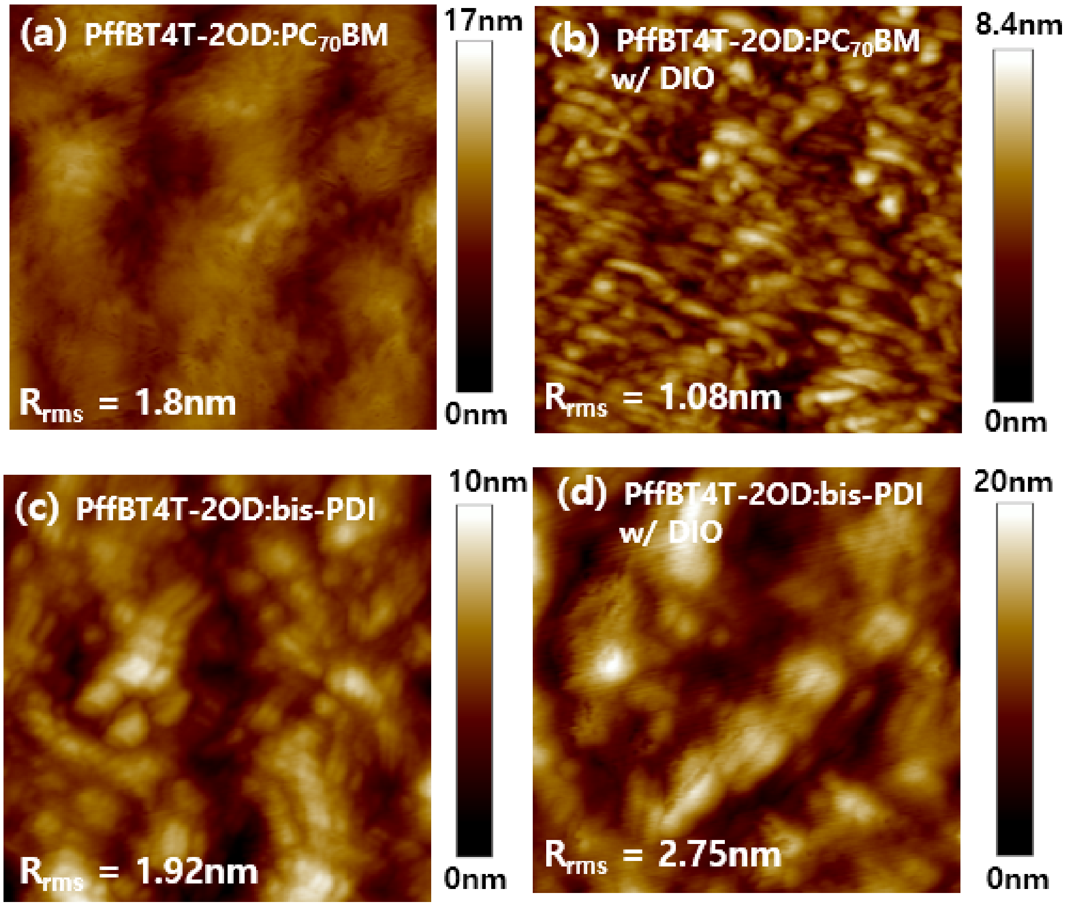

Figure S4. AFM height images for the blend films of (a) PffBT4T-2OD:PC_{70}BM, (b) PffBT4T-2OD:PC_{70}BM w/DIO, (c) bis-PDI, PffBT4T-2OD:bis-PDI and (d) PffBT4T-2OD:bis-PDI w/DIO, where scan area is 1 × 1 μm^2.

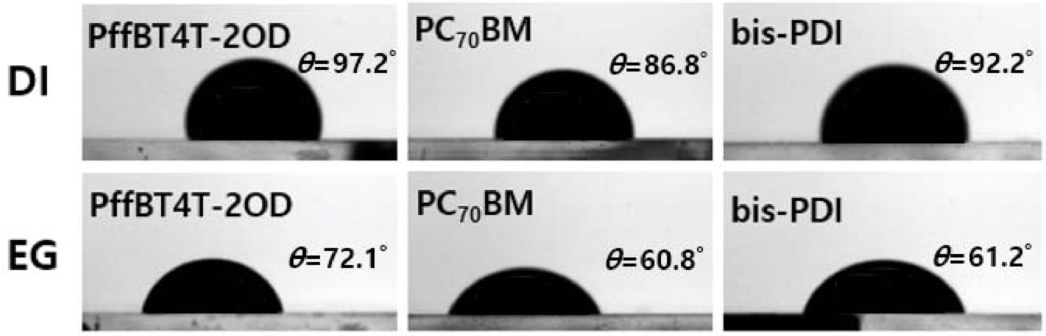

Figure S5. Contact angle (θ) values of the pristine PffBT4T-2OD, $PC_{70}BM$ and bis-PDI with respect to two solvents, DI and EG.

Table S3. Contact angle (θ) and surface energy (γ) values of the pristine PffBT4T-2OD, $PC_{70}BM$ and bis-PDI. The contact angle value obtained by averaging three contact angles measured at three different positions of the sample. The surface energy of the film was calculated by the measurement of the contact angle made between the film and two different liquids (DI and EG). The contact angles were entered in the Owens and Wendt geometric mean equation for the calculation of surface energy.[7]

Films	$\theta_{DI\ water}$ (°)	θ_{EG} (°)	$\gamma_{dispersive}$ (mNm^{-1})	γ_{polar} (mNm^{-1})	γ_{total} (mNm^{-1})
PffBT4T-2OD	97.2	72.1	28.9	0.23	29.1
$PC_{70}BM$	86.8	60.8	29.5	2.1	31.6
bis-PDI	92.2	61.2	41.1	0.014	41.1

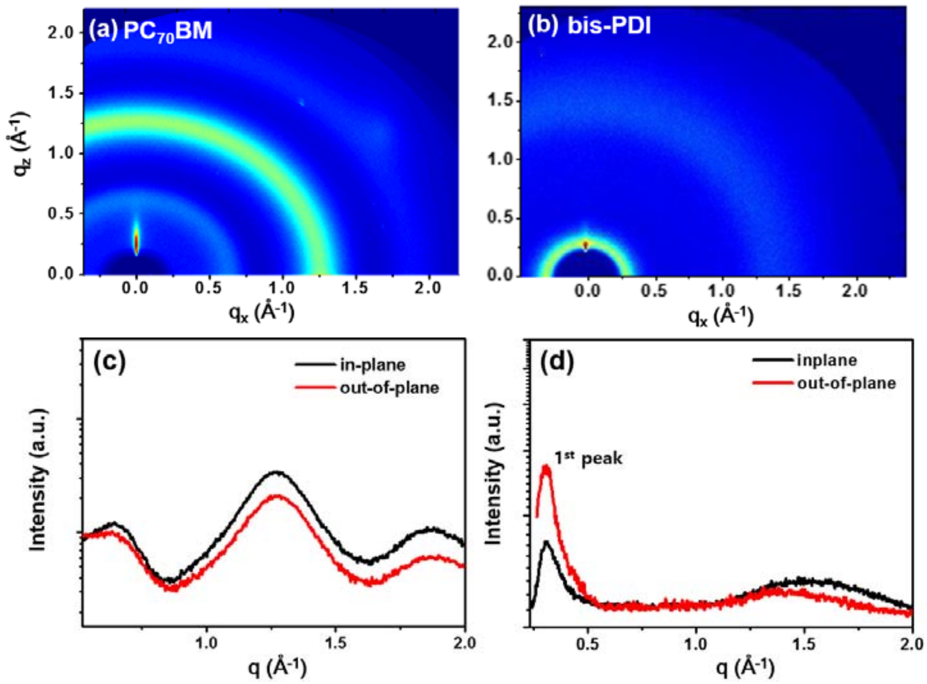

Figure S6. (a & b) GIWAXS images and (c & d) in-plane and out-of-plane scan for the as-spun $PC_{70}BM$ and bis-PDI neat thin films.

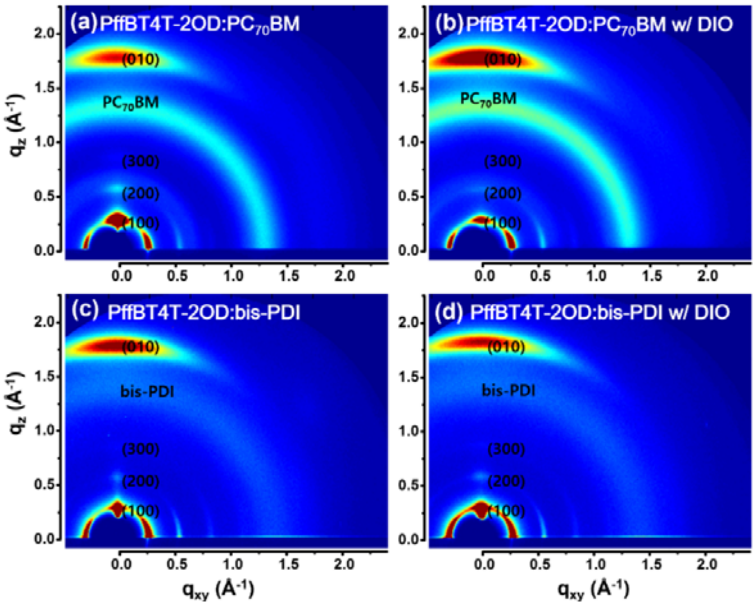

Figure S7. GIWAXS images of the blend films of (a) PffBT4T-2OD:$PC_{70}BM$, (b) PffBT4T-2OD:$PC_{70}BM$ w/DIO, (c) PffBT4T-2OD:bis-PDI and (d) PffBT4T-2OD:bis-PDI w/DIO.

Table S4. GIWAXS results for PffBT4T-2OD:$PC_{70}BM$ and PffBT4T-2OD:bis-PDI the blend films w/o DIO and w/DIO additive.

D:A		Peak Index	Peak Position ($Å^{-1}$)	Spacing (Å)	Coherence Length (Lc) (Å)
PffBT4T-2OD:$PC_{70}BM$	in-plane	200	0.575	10.92	117.55
	Out-of-plane	010	1.747	3.60	39.44
PffBT4T-2OD:$PC_{70}BM$ w/DIO	in-plane	200	0.570	11.02	139.93
	Out-of-plane	010	1.739	3.61	43.38
PffBT4T-2OD:bis-PDI	in-plane	200	0.567	11.07	155.41
	Out-of-plane	010	1.741	3.61	44.91
PffBT4T-2OD:bis-PDI w/DIO	in-plane	200	0.562	11.17	158.47
	Out-of-plane	010	1.758	3.57	49.70

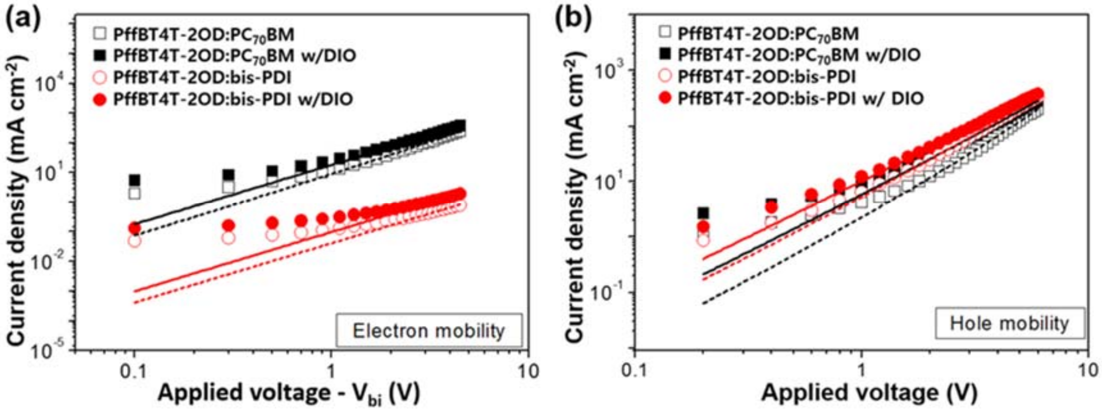

Figure S8. Dark current density versus effective voltage characteristics of (a) electron-only devices and (b) hole-only devices, with photoactive layers PffBT4T-2OD:$PC_{70}BM$, PffBT4T-2OD:bis-PDI. The dotted lines are fits based on Mott-Gurney equation.[6]

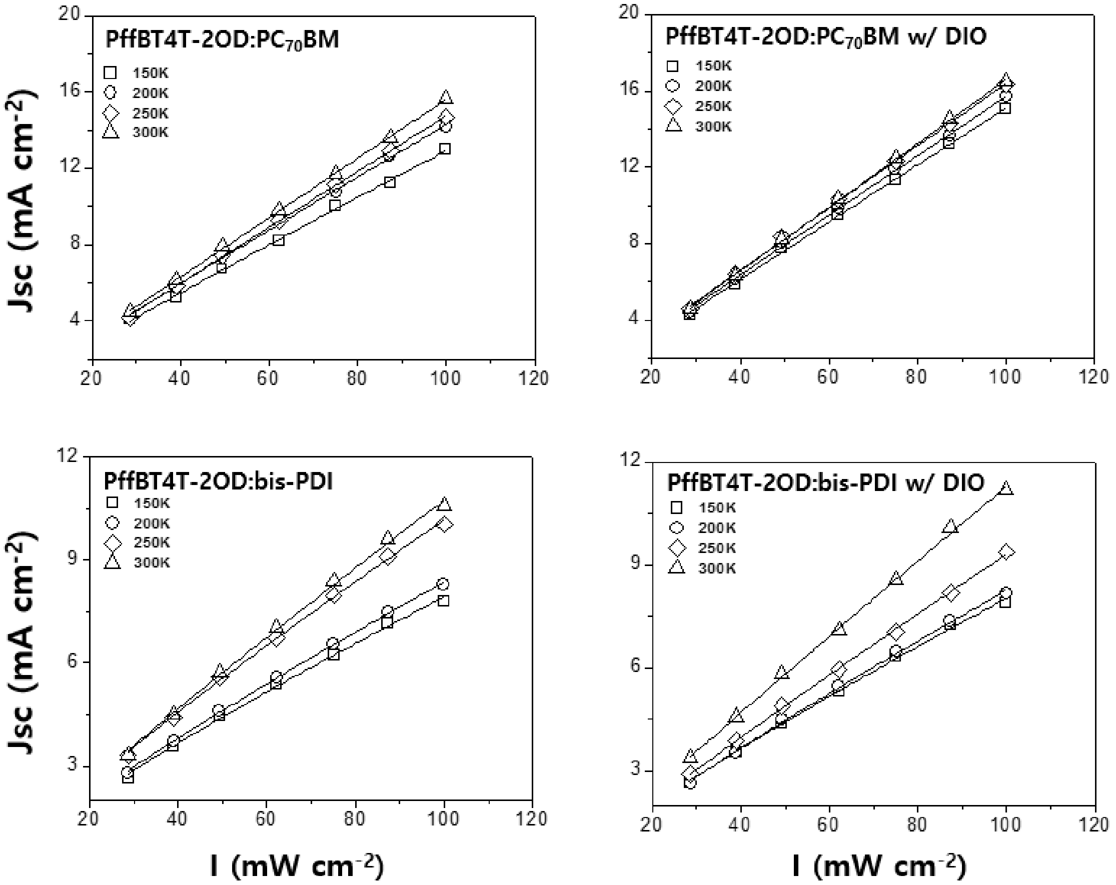

Figure S9. Light intensity (I) dependence current density (J_{SC}) for PffBT4T-2OD:PC_{70}BM, PffBT4T-2OD:bis-PDI w/o DIO and w/DIO based OSCs under different temperatures. Solid lines show the fitting for the experimental data with equation $J_{SC} \propto I^{\alpha}$.[8]

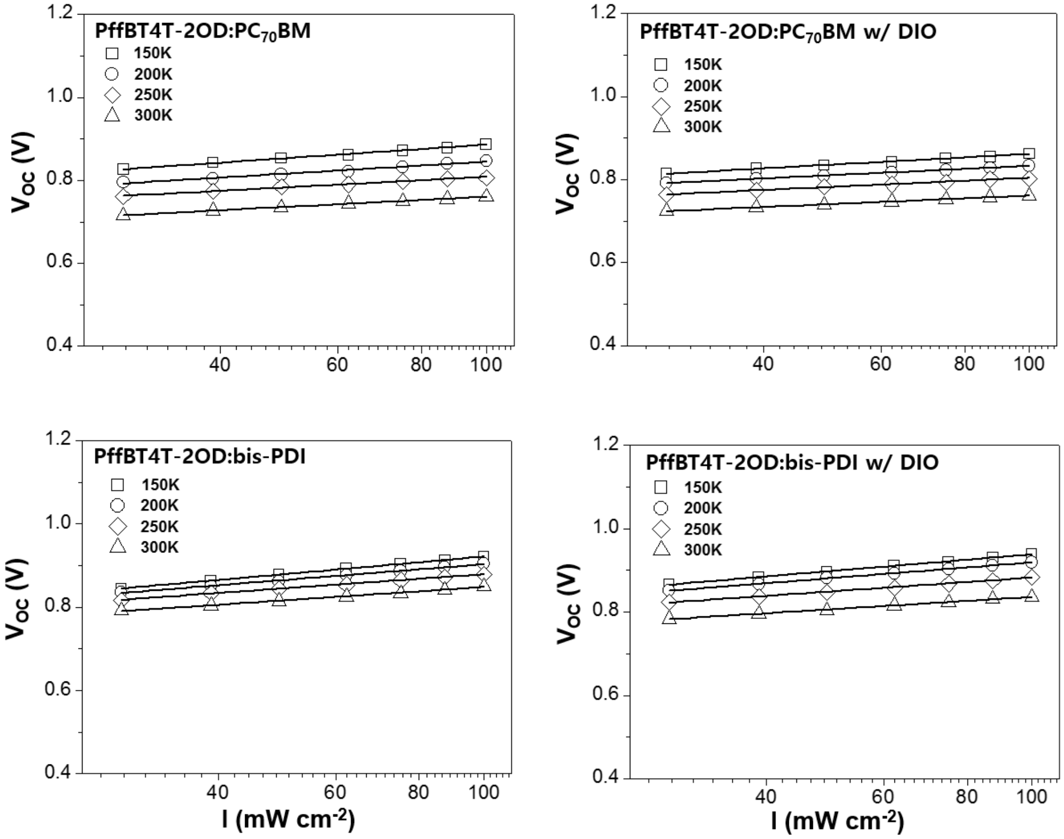

Figure S10. Light intensity (I) dependence open-circuit voltage (V_{OC}) for PffBT4T-2OD:$PC_{70}BM$, PffBT4T-2OD:bis-PDI w/o DIO and w/DIO based OSCs under different temperatures. Solid lines show the fitting for the experimental data with equation $V_{OC} \propto \beta(K_BT/e)\ln(I)$.[8]

Table S5. The width of the density of states σ_h and σ_e calculated from temperature-dependent elctron and hole mobilties plot in Figure 7.

D:A	σ_h (meV)	σ_e (meV)
PffBT4T-2OD:$PC_{70}BM$	62.0	74.6
PffBT4T-2OD:$PC_{70}BM$ w/DIO	51.7	67.9
PffBT4T-2OD:bis-PDI	38.8	81.5
PffBT4T-2OD:bis-PDI w/DIO	34.6	79.2

References

1. Y. Huang; J. Hu; W. Kuang; Z. Wei; C. F. Faul, *Chemical Communications* **47**, 5554, (2011).
2. Q. Yan; D. Zhao, *Organic Letters*, **2009**, 11, 3426.
3. W. Jiang; L. Ye; X. Li; C. Xiao; F. Tan; W. Zhao; J. Hou; Z. Wang, *Chem. Commun.s* **50**, 1024 (2014).
4. Q. F. Yan, Y. Zhou; Y. Q. Zheng; J. Pei; D. H. Zhao, *Chem. Science* 4, 4389 (2013).
5. J. Pommerehne; H. Vestweber; W. Guss; R. F. Mahrt; H. Bässler; M. Porsch; J. Daub, *Adv.d Mater.* **7**, 551 (1995).
6. T. Ye; R. Singh; H. -J. Butt; G. Floudas; P. E. Keivanidis, *ACS Appl. Mater. Interfaces* **5**, 11844 (2013).
7. Owens, D. K.; Wendt, R. C., *J. of App.Polymer Science 13,* 1741-1747 (1969).
8. S. R. Cowan; A. Roy; A. J. Heeger; *Phys. Rev. B* **82**, 245207 (2010).
